# A Perspective on the Similarities and Differences Between Mindfulness and Relaxation

**DOI:** 10.1177/2164956120905597

**Published:** 2020-02-05

**Authors:** Christina M Luberto, Daniel L Hall, Elyse R Park, Aviad Haramati, Sian Cotton

**Affiliations:** 1 Benson-Henry Institute for Mind-Body Medicine, Massachusetts General Hospital/Harvard Medical School, Boston, Massachusetts; 2 Mongan Institute Health Policy Research Center, Massachusetts General Hospital/Harvard Medical School, Boston, MA; 3 Department of Biochemistry, Molecular and Cellular Biology, Georgetown University School of Medicine, Washington, District of Columbia; 4 Department of Family and Community Medicine, University of Cincinnati College of Medicine, Cincinnati, Ohio

**Keywords:** mindfulness, relaxation, health, well-being, mind–body

## Abstract

Mind–body medicine is an evidence-based approach to health and healing that focuses on interactions between the mind, body, and behavior. It encompasses a wide range of interventions that are similar yet different in meaningful ways. Mindfulness and relaxation practices are 2 mind–body techniques that have similarities and differences; however, these techniques are often used or discussed interchangeably, such that the differences between them become obscured. A greater understanding of the unique facets of mindfulness and relaxation is needed for researchers and clinicians to make informed decisions when selecting an approach. The purpose of the current article is to offer an evidence-informed perspective on similarities and differences between mindfulness and relaxation. Specifically, mindfulness and relaxation practices are compared and contrasted in terms of theoretical foundation, intention, and psychological and physiological effects and mechanisms. Implications for clinical practice and suggestions for future research are discussed.

## Introduction

Mind–body medicine is an evidence-based healing approach that focuses on the interactions between the mind, body, and behavior.^1^ It encompasses a diverse range of techniques that all aim to unite the mind and body to promote health and well-being.^1^ Examples include meditation (eg, mindfulness meditation, loving kindness meditation, transcendental meditation), relaxation practices (eg, diaphragmatic breathing, progressive muscle relaxation, guided imagery), and meditative movement practices (eg, yoga, tai chi).^1^ This variety allows clinicians, researchers, and practitioners to select a specific approach best suited to their interests and needs, with the knowledge that each one could benefit health and well-being by uniting the body and mind.

There are also key differences among mind–body approaches that are important for clinicians and researchers to consider. In particular, it is important to consider the similarities and differences between mindfulness meditation and relaxation practices, given their significant overlap and the widespread use. Establishing clear distinctions between these approaches poses several challenges: many mind–body interventions are multimodal programs that combine aspects of both approaches, and both approaches have overlapping components (as discussed in detail later). At the same time, the mindfulness literature highlights that mindfulness and relaxation are different techniques^2–5^ and the National Center for Complementary and Integrative Health (NCCIH) discusses meditation practices in a separate category from relaxation practices, while noting that there is in fact some overlap.^1^

Despite the acknowledgment of their differences, mindfulness and relaxation approaches are often used or discussed interchangeably by researchers and clinicians such that the differences between them become obscured. For example, relaxation techniques are often used as the control group in mindfulness-based intervention research,^6^^,^^7^ demonstrating that researchers consider these 2 approaches to be meaningfully different, but clinicians often use mindfulness practices to elicit calm or relaxation for patients, suggesting that many see these approaches as interchangeable. Scientific study of the similarities and differences between mindfulness and relaxation is limited, particularly in terms of their mechanisms and effects on outcomes. In practical terms, there is a need for greater attention to compare and contrast mindfulness and relaxation approaches in order to design mind–body interventions with appropriate control groups and deliver each technique thoughtfully in clinical practice.^6^^,^^8^

The purpose of the current article is to offer a narrative overview of the similarities and differences between mindfulness and relaxation to help guide future mind–body medicine research and clinical practice. We first provide definitions and brief backgrounds for mindfulness and relaxation practices and describe the physiology of the stress response and relaxation response (RR) to inform this discussion. We next review the similarities between mindfulness and relaxation practices and then review their differences in terms of (1) cognitive-behavioral theories regarding health improvement, (2) the intentions of mindfulness and relaxation practices, and (3) illustrative research findings regarding potential differences in psychological and physiological outcomes and mechanisms. Lastly, we discuss implications for future research and offer evidence-based suggestions for clinical practice.

## Definitions and Backgrounds

### Mindfulness

Mindfulness meditation is a 2500-year-old practice grounded in Eastern philosophical and ethical traditions, particularly vipassana meditation practices of Buddhism. It is derived from the Pali word *sattipathana*, which loosely translates to “remembering.” Several resources on the Buddhist origins of mindfulness are available for further background.^9–11^ The focus of the current article is on the use of secularized mindfulness practices as applied in clinical settings.

Mindfulness is commonly defined as “the awareness that emerges through paying attention in a particular way: on purpose, in the present moment, and nonjudgmentally.”^12^ It is essentially a “way of being” that involves intentionally self-regulating one’s attention toward current moment experiences, and noticing those experiences with an attitude of openness, acceptance, and curiosity; the opposite of “automatic pilot.”^13^^,^^14^ The nonjudgmental quality of mindful attention is important for developing an accepting attitude toward present moment events. Mindfulness can refer to a state (ie, being mindful in a given moment), a trait (ie, the tendency to be mindful in everyday life), or mindfulness meditation practices. The overall goal of mindfulness training in clinical settings is to develop a more open, nonreactive relationship with internal experiences (eg, thoughts, emotions, physical sensations) through engagement in mindfulness practices in order to reduce suffering and promote adaptive behaviors.

### Mindfulness Practices

Mindfulness (as a state or trait) can be cultivated through a variety of formal and informal mindfulness practices.^15^^,^^16^ All mindfulness practices involve setting an intention to focus on a specific “object of awareness” happening in the present moment and maintaining and redirecting attention toward that object whenever the mind wanders; this includes noticing when attention has drifted from the chosen object and gently guiding it back. These “objects” can include various internal or external sensory experiences such as the breath, body sensations, thoughts, or sounds. A key element of mindfulness practices is noticing experiences with openness and curiosity, without trying to change or suppress them.

Formal mindfulness practices are those that involve taking time out of the day, typically anywhere from 5 to 40 minutes, to practice noticing chosen objects of awareness openly and with non-judgment. Examples include the body scan (eg, openly noticing physical sensations throughout the body), certain seated meditations (eg, maintaining attention to openly notice the breath, body sensations, sounds, thoughts, and emotions), and movement-based meditations. Advanced mindfulness practices can also include choice-less awareness/open monitoring, wherein individuals do not intentionally direct attention toward any specific object but instead practice openness and nonattachment to all events that flow through awareness, essentially meditating on awareness itself (ie, meta-awareness).

Informal mindfulness practices include any daily activity during which individuals intentionally attempt to bring undivided, mindful attention, such as mindful walking or eating. The general instructions are the same: attempting to maintain focused, open attention on the activity at hand and gently redirecting attention back to the activity when the mind wanders off. The difference is that informal practices do not take additional time out of the day and may be practiced organically during a chosen activity.

### The Relaxation Response

First identified by Dr Herbert Benson of Harvard Medical School through research on transcendental meditation in the 1970s, the RR refers to a physiological state of parasympathetic dominance; that is, a physiological state in which there is greater activation of the parasympathetic nervous system in relation to the sympathetic nervous system. The parasympathetic and sympathetic nervous systems are 2 branches of the autonomic nervous system which work alongside one another to initiate, sustain, or dampen a variety of physiological functions throughout the body. The parasympathetic nervous system is colloquially known as the “rest and digest” system, whereas the sympathetic nervous system is chiefly involved in the stress response, also known as the “fight or flight response.”

**Figure 1.**
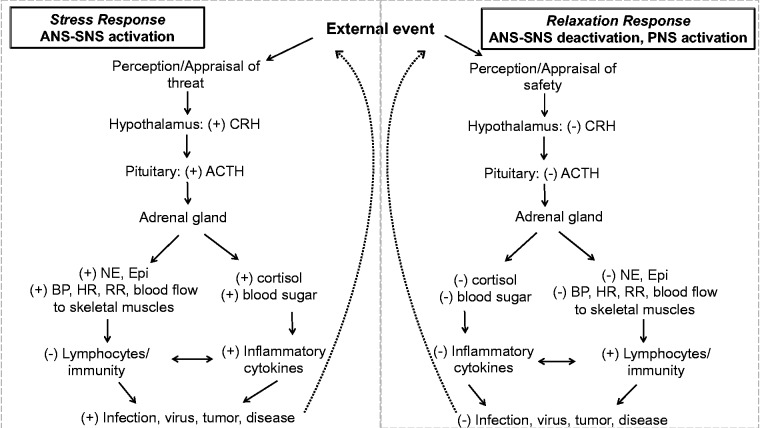
Overview of the physiology of the stress response and relaxation response. ACTH, adrenocorticotropic hormone; ANS, autonomic nervous system; BP, blood pressure; CRH, corticotrophin-releasing hormone; HR, heart rate; NE, norepinephrine; PNS, parasympathetic nervous system; RR, relaxation response; SNS, sympathetic nervous system.

Figure 1 depicts an outline of parasympathetic and sympathetic nervous systems. Briefly, both systems can be triggered by one’s perception of whether an external event poses a potential threat. Qualities of external stimuli that most reliably trigger the sympathetic nervous system include (1) uncontrollability and (2) socioevaluative threat (eg, being judged or evaluated).^17^ If the sympathetic nervous system is activated, corticotrophin-releasing hormone (CRH) is released, which stimulates the pituitary to release adrenocorticotropic hormone (ACTH). ACTH then stimulates the adrenal cortex to release glucocorticoids (ie, cortisol) and the adrenal medulla to secrete catecholamines (ie, adrenaline).^18^ Glucocorticoids and catecholamines both initiate physiological states commonly associated with stress; for instance, glucocorticoids raises blood sugar and the catecholamines have cardiovascular effects to increase blood pressure, heart rate, cardiac output, and blood flow to skeletal muscles. These changes typically return back to baseline after the resolution of the stressful event, but much depends on what other challenges are being addressed. Furthermore, the ability to respond appropriately depends on many factors including the physical fitness of the individual, epigenetic factors (ie, trauma exposure), and health-related behaviors. What is also clear is that elevated stress hormone levels compromise the ability of an individual to respond to novel stressors adequately. Researchers have also found that a chronically activated sympathetic nervous system may raise one’s baseline levels of arousal—a phenomenon termed “allostatic load”—thereby predisposing them to exaggerated physiological responses when exposed to future stressors.^19^ In turn, a high allostatic load significantly increases the risk of physical and mental health problems,^18^ accounting for up to 80% of primary care visits.^20^^,^^21^

In contrast, if the event is not perceived as a threat, the parasympathetic nervous system is stimulated, thereby engaging the RR. As depicted in Figure 1, the RR is characterized by a blocking of the physiological processes associated with the stress response (ie, CRH, ACTH, cortisol, and the catecholamines are not released). Thus, physiological changes such as decreases in heart rate, blood pressure, and respiration rate are indicative of the RR. Elicitation of the RR has been shown to improve dysregulated hypothalamic–pituitary–adrenal axis functioning in a wide array of chronic illness populations.^22–27^ In turn, the RR can lead to acute and persistent decreases in blood pressure, heart rate, and respiration rate. In this way, the RR can promote restoration and healing of the body and mind by decreasing the physiological and psychological burden of chronic stress.^28^

### Relaxation Practices

The RR can be elicited through engagement in various mind–body practices, including both relaxation practices as well as mindfulness practices (described in more detail later).^29^^,^^30^ Typically, to elicit the RR, individuals aim to develop a single point of focus by repeating a word or phrase (eg, a mantra, counting) and adopting a passive attitude to set aside intrusive thoughts or images, while sitting quietly in a comfortable position and aiming to relax the body and mind.^29^ Thus, single-pointed meditation, which involves maintaining attention on one focal point (eg, the breath, a mantra), can be considered the cornerstone of the RR.^31^

Other examples of formal RR elicitation practices include diaphragmatic breathing, guided imagery, and progressive muscle relaxation. Each of these practices involve the intention and effort to create new physical or emotional states of increased calm and decreased arousal, often by slowing the breath and/or generating new physical or mental experiences. Diaphragmatic breathing involves intentionally slowing and deepening the breath to decrease arousal. Guided imagery practices use mental visualizations to elicit relaxation, often in combination with slowed diaphragmatic breathing. Progressive muscle relaxation is another technique that involves intentionally tensing and releasing various muscles groups to generate feelings of tension release and relaxation.^32^

Relaxation can also be elicited during everyday activities. Any activity during which individuals maintain undivided attention and experience parasympathetic dominance can be considered a relaxation practice, and these likely vary across individuals. For example, some people may experience gardening to be relaxing, while others experience relaxation during cooking, cleaning, or reading.

## Similarities

There are several similarities between mindfulness and relaxation practices. First, both types of practices can elicit the RR to offset the stress response. Individuals have reported decreased stress and tension, with corresponding physiological changes that indicate parasympathetic activation (eg, reduced heart rate, respiration rate), following relaxation practices^33–36^ and mindfulness practices.^37–43^ Thus, both approaches can improve health outcomes by offsetting the negative health effects of chronic stress and reducing symptoms of stress-related health problems. Indeed, both types of practices have evidence of efficacy for treating similar problems. Results of randomized controlled trials (RCTs) suggest that interventions focused on both mindfulness and relaxation practices can improve health outcomes including stress, anxiety, depression, and pain.^44–48^

Second, many mind–body practices and interventions are multi-modal and incorporate elements of both mindfulness and relaxation. For example, certain yoga practices include RR elicitation components (eg, savasana) and also encourage awareness and approaching of discomfort during the same yoga class. There are also evidence-based mind–body interventions based on the RR that include mindfulness practices.^49^ Multimodal mind–body interventions that incorporate both mindfulness and relaxation practices have shown feasibility and efficacy for improving a range of health outcomes.^48^^,^^50–54^

Third, to the extent that individuals attempt to maintain focus on present moment experiences during relaxation practices, and notice these experiences with openness and acceptance, relaxation practices involve mindful awareness. There is some preliminary evidence to suggest that trait mindfulness may increase with participation in relaxation-based interventions.^55^ Experimental studies assessing changes in state mindfulness are mixed. The results of one study suggested that brief mindfulness and relaxation practices had similar effects on cognitive decentering (the attentional component of mindfulness),^6^ while another study suggested that mindful breathing produced greater increases in cognitive decentering as compared to progressive muscle relaxation.^56^ Further research is needed to clarify the effects of relaxation practices on mindful awareness and should include assessments of both state and trait mindfulness, following both brief, single-session relaxation practices and longer relaxation-based intervention packages.

Lastly, mindfulness and relaxation practices share common elements and may be used in tandem to enhance the other. Mindfulness can promote the effective use of relaxation practices by helping people become aware of whether or not behaviors that are intended as relaxation are actually relaxing. For example, an individual might watch TV or use social media with the intention to relax, but with greater awareness realize that their body is actually tense and tight, and that they feel anxious or frustrated during these activities. Another common element is that both mindfulness and relaxation can be practiced formally and informally. Mindfulness can be practiced informally via focused awareness of an everyday activity, and everyday activities that elicit the RR can be considered informal relaxation practice. In terms of formal practices, both practices can incorporate mental imagery. Examples include guided imagery RR elicitation practices and mindfulness meditation practices that employ images as a way to promote awareness and non-attachment of thoughts (eg, visualizing thoughts as leaves floating down a stream).

Given these areas of overlap, the discussion of differences between mindfulness and relaxation is not a question of which approach is “better.” The importance of understanding differences between these 2 approaches relates more to determining which approach is most aligned with a particular clinical situation (eg, what works best for whom, and when), or which approach is best suited to answer a specific research question.

## Differences

### Theoretical Foundation

Table 1 provides a summary of the differences between mindfulness and relaxation. In terms of theoretical foundation, there are some differences in how mindfulness and relaxation are thought to improve health. Although mindfulness practices can elicit the RR, and the reduction of chronic stress is one rationale for applying mindfulness practices in clinical settings, cognitive-behavioral theories from clinical psychology suggest that mindfulness training may also improve psychological outcomes through mechanisms that do not depend on the RR. These theories suggest that the benefits of mindfulness practice for psychological health may be facilitated by approaching the discomfort that can arise during mindfulness meditation practices. The history of cognitive-behavioral therapy (CBT) illustrates different theoretical rationales for applying relaxation and mindfulness practices.

**Table 1.** Summary of Differences Between Mindfulness and Relaxation.

Mindfulness	Relaxation
Theoretical foundation	
Third-wave CBT approach	Traditional CBT approach
Automatic reaction patterns can contribute to suffering	Chronic stress and/or an overactive stress response cause disease
Experiential avoidance maintains psychological rigidity, exposure fosters flexibility	Eliciting the relaxation response counters the stress response to reduce chronic stress
Taking thoughts as facts can be problematic, cognitive de-centering undermines dominance of discursive thinking	States of relaxation provide access to psychological resources that build resiliency to improve health outcomes
Intention of practices	
Noticing present moment events as they are facilitates conscious choice	Elicit parasympathetic dominance
Emphasis on accepting present moment internal events to reduce struggle with own thoughts and feelings	Emphasis on changing present moment internal events
Examples of types of practices	
Body scan (notice physical sensations as they naturally occur throughout the body, raising interoceptive awareness and reactions to discomfort)	Progressive muscle relaxation (purposefully relax muscles by systematically tensing and releasing specific muscle groups)
Awareness of breath (notice the breath as it naturally occurs, redirect attention back to the breath when it wanders, letting go of attempts to control automatic processes)	Deep breathing (purposefully slow and deepen the breath)
Sitting meditation (sequentially notice internal and external events such as sounds, thoughts, raising awareness of automatic patterns of thinking and reacting)	Guided imagery (generate imagined scenarios)
Psychological outcomes and mechanisms	
Improvements in mood, stress and general well-being	Improvements in mood, stress, and general well-being
Improvements in nuanced emotional processes, including following brief, single-session practices	Potential improvements in nuanced emotional processes but usually only after multi-session interventions

#### Traditional CBT: emphasis on relaxation practices

CBT is the gold-standard evidence-based nonpharmacological treatment approach for managing problems such as anxiety, depression, and poor health behaviors.^57^^,^^58^ CBT is a short-term, active, skills-based treatment that engages patients in learning and applying practical skills for working with difficult thoughts, emotions, and behaviors. Initially, CBT focused on directly changing individuals’ thoughts, emotions, physical sensations, and behaviors using strategies such as cognitive restructuring, behavioral experiments, and relaxation techniques.^59^ Thus, CBT is not typically considered a “relaxation intervention” per se, as it incorporates a variety of skills and different clinicians may incorporate relaxation practices to greater or lesser extents—but of the different mind–body practices, traditional CBT emphasized relaxation practices as a way to change uncomfortable internal experiences. Examples of evidence-based mind–body interventions that emphasize relaxation training and traditional CBT principles include the Stress Management and Resiliency Training—Relaxation Response Resiliency Program (SMART-3RP)^49^ and Cognitive-Behavioral Stress Management (CBSM).^60^ These interventions are typically delivered over 8 weekly 1- to 2-hour sessions in a group format and include didactic components as well as experiential relaxation practices.

In the late 1990s and early 2000s, controversial findings emerged that attempts to directly change internal experiences were not needed for patients to improve. Clinical trial results did not indicate that cognitive change was an active mechanism of CBT and behavioral interventions that did not include cognitive change strategies still had a large and significant benefit for improving emotional problems.^61–63^ There were also concerns that attempts to directly change internal experiences could reinforce the same psycho-behavioral processes that caused the emotional problem in the first place; namely, a need to avoid or change uncomfortable internal experiences (ie, experiential avoidance).^64^ As a result of these research findings and clinical concerns, a new “third-wave” CBT paradigm emerged that emphasized changing the way individuals relate to internal experiences, rather than changing the actual form or intensity of the experience itself.^64^

#### Third-wave CBT: emphasis on mindfulness practices

Third-wave CBT interventions emphasize mindfulness practices over relaxation practices to help patients develop open, nonjudgmental, and nonreactive relationships to internal experiences. The goal of these interventions is to help patients learn that internal experiences such as thoughts, emotions, and body sensations may be uncomfortable but are typically harmless and thus do not necessarily need to be changed or eliminated in order to live a meaningful life. Moreover, the theory is that it is actually the attempts to change or avoid these internal experiences that transform harmless emotions into functionally impairing emotional disorders.^64^

Core components of mindfulness training as taught in third-wave CBT include present moment awareness, attention regulation, and a nonjudgmental attitude. Theoretically, these skills promote improvements in cognitive-affective processes that underlie emotional and behavioral problems.^65^ For example, through present moment awareness and nonreactivity of discomfort, mindfulness practices function as exposure exercises to help individuals improve their ability to tolerate emotional distress.^14^^,^^66^ Mindfulness can also strengthen attention regulation skills and a present moment orientation to interrupt repetitive thinking patterns (eg, worry and rumination) that characterize emotional disorders (eg, major depressive disorder, generalized anxiety disorder) as well as reduce the significance of unhelpful thoughts by promoting cognitive de-centering, which refers to the ability to view thoughts as transient mental events separate from the self.^67^ As noted earlier, these improvements do not depend on individuals being in a physiologically relaxed state and, in fact, may be maximized in the absence of relaxation.

Examples of third-wave CBT interventions that emphasize mindfulness include Mindfulness-Based Stress Reduction (MBSR),^68^ Mindfulness-Based Cognitive Therapy (MBCT),^69^ Dialectical Behavior Therapy (DBT),^70^ Acceptance and Commitment Therapy (ACT),^71^ and Acceptance-Based Behavior Therapy (ABBT).^72^ MBSR and MBCT are among the most well-known mindfulness-based interventions. They follow a similar structure (8 weekly 1- to 2-h sessions delivered in a group format), though MBCT takes a more explicit CBT approach as compared to MBSR.

### Intentions of Mindfulness and Relaxation Practices

The intention of mindfulness and relaxation practices differ in ways that align with the cognitive-behavioral theoretical foundations described earlier. The goal of mindfulness practices in clinical settings is to build awareness and acceptance skills in order to tolerate discomfort, gain distance from unhelpful thoughts, and ultimately make adaptive behavioral choices to reduce suffering and pursue valued goals—even while uncomfortable emotions or physical sensations may be present. The goal of relaxation practices is to elicit the RR to directly reduce physiological and psychological stress, decrease physical tension, and increase a sense of calm in order to promote positive health behaviors and outcomes.^73^^,^^74^ In short, mindfulness practices teach acceptance of present moment internal events, while relaxation practices teach strategies to change internal events.

An example of how mindfulness and relaxation practices can be taught in clinical settings may help illustrate these differences in intention. For example, for a patient with anxiety, an RR elicitation practice such a progressive muscle relaxation could be used to teach the patient how to decrease anxiety and relax the body, if the clinician’s goal is to help the patient minimize physical and cognitive anxiety symptoms. Here, the patient would be taught skills to change the internal state of the body to elicit a feeling of calm. If the clinician’s goal was to teach the patient how to tolerate anxiety symptoms by sitting with them, noticing them openly, and allowing them to pass naturally, then a mindfulness practice such as the body scan may be used instead. In this case, the patient would be learning skills to withstand anxiety symptoms so that these symptoms do not need to get in the way of engaging in valued activities.

Thus, the differences in intention generally come down to accepting internal events (mindfulness) and changing internal events (relaxation). We view these differences as complementary rather than contradictory and understand that mindfulness and relaxation practices may often be taught together. The key point is to be aware of the differences in the intentions of these practices and select and frame each one carefully in an appropriate way. For example, each approach may be used at a different time depending on the patient’s needs and goals (ie, whether acceptance or change skills are more appropriate for the situation). We provide further suggestions for clinical practice in the Clinical Implications section later.

### Psychological Outcomes and Mechanisms

Research supports the efficacy of both mindfulness and relaxation training for improving psychological and physical health outcomes across a variety of patient populations.^48^^,^^50^^,^^75^^,^^76^ Multiple meta-analyses of mindfulness-based interventions support improvements in stress, mood, anxiety, and pain.^44,77,78^ Research similarly suggests that relaxation techniques may be helpful for anxiety associated with medical procedures or conditions, insomnia, labor pain, chemotherapy-induced nausea, and joint dysfunction.^1^ Research on differential effects of these 2 approaches is mixed.

Several RCTs and experimental studies have compared mindfulness and relaxation training for their effects on psychological processes. Many studies have found significant differences between these approaches. Sedlmeier et al.^79^ conducted a systematic review of meditation for psychological health, which included 10 RCTs that compared mindfulness to relaxation. The authors concluded that mindfulness and relaxation share commonalities but are different practices, with mindfulness showing larger effect sizes for most psychological outcomes (eg, well-being, anxiety, stress).^79^ In line with the theoretical foundation described earlier, other studies have found differential effects favoring mindfulness training in terms of emotional tolerance processes (eg, cognitive de-centering, rumination, emotional reactivity).^7,56,80–82^ For example, Feldman et al.^56^ randomly assigned novice meditators to a 15-minute mindful breathing, progressive muscle relaxation, or loving kindness meditation practice via audio recording. The results indicated that the association between repetitive thoughts and negative reactions to thoughts was weaker in the mindfulness condition than the other 2 conditions, suggesting that mindfulness uniquely decreases emotional reactivity and promotes cognitive decentering.^56^ In an RCT of a 1-month mindfulness intervention compared to a relaxation intervention, Jain et al.^7^ found that while both interventions reduced distress and increased positive mood relative to a no-treatment control, mindfulness meditation uniquely reduced repetitive thinking. Another study similarly found that as compared to relaxation practice, mindfulness practice was associated with improved real-time regulation of distress, as evidenced by lower distress levels during a stressful laboratory task among young adults with chronic pain.^83^

Other research, however, has found comparable improvements in emotional tolerance with the use of both approaches. For example, in a recent RCT comparing relaxation to a multimodal mindfulness-based intervention (acceptance-based behavioral therapy), both produced similar improvements in anxiety and depression symptoms,^8^ cognitive de-centering^84^ and experiential avoidance.^85^ Moreover, changes in cognitive decentering and experiential avoidance served as mechanisms of effects on anxiety symptoms in both treatment groups.^84,85^ One difference between this trial and some of those that found differential improvements is the length of the mindfulness and relaxation training: here, participants received at least 8 weekly sessions, ranging from 60 to 90 minutes each,^8^ while in other studies, differential effects were shown after shorter trainings (eg, single 10–15 min mindfulness exercises or 4-week interventions).^7,56,82^ Similarly, in an RCT comparing an acceptance-based intervention to progressive muscle relaxation, the acceptance group showed greater improvements in experiential avoidance at postintervention, but both groups showed comparable improvements at 3-month follow-up.^86^ This research raises the question of whether mindfulness and relaxation can ultimately produce similar effects on emotional tolerance processes depending on the dose of practice, and more research is needed to understand dose effects.

### Physiological Outcomes and Mechanisms

As mentioned earlier, both mindfulness and relaxation practices can promote physiological changes associated with the RR. Both practices have also been associated with improvements in stress-related physiological outcomes such as hypertension, immune function, genomic effects on inflammatory markers, mitochondrial function, insulin secretion, telomere maintenance, decreased expression of genes linked to inflammatory response and stress pathways, and reduced pro-inflammatory cytokines.^87–89^

Direct comparisons between mindfulness and relaxation on physiology are also emerging. The results of several studies have shown differences between the 2 approaches. Controlling for attention, demand, and practice effects using a “sham” mindfulness meditation control group focused on deep breathing, one study found that mindfulness meditation yielded unique cardiovascular benefits (eg, reduced heart rate).^42^ Another RCT compared MBSR to progressive muscle relaxation and found greater improvements in blood pressure following MBSR.^90^ Recently, researchers compared neural activity following a relaxation-based intervention (SMART-3RP) and mindfulness-based intervention (MBSR) and found both overlapping and dissociable patterns of activation, where differences aligned with each program’s theoretical focus described here.^91^ In an RCT of yoga as compared to a multimodal mind–body intervention (CBSM), yoga was found to produce greater improvements in muscle strength.^92^ Further research is needed to compare physiological and neurological effects of mindfulness and relaxation before firm conclusions can be drawn.

### Implications for Clinical Practice

Given that mindfulness and relaxation training are both useful for improving physical and emotional health outcomes, clinicians might select either approach or use a combination of approaches when treatment goals involve general well-being or stress management, with the primary consideration being patient preferences or abilities. Relaxation practices may seem more familiar and concrete than mindfulness practices to some individuals, such as those with concrete thinking styles, psychiatric vulnerabilities, or cognitive impairments. Mindfulness practices may offer unique benefits for patients with certain emotional disorders, namely those that involve experiential avoidance and/or repetitive thinking (eg, generalized anxiety disorder, major depressive disorder). For example, mindfulness may have advantages for individuals with panic disorder who tend to fear innocuous body sensations and could benefit from learning to allow physical symptoms to be as they are, without the need to change them through a relaxation approach. However, the evidence to date is mixed, and it is possible that relaxation techniques can produce the same improvements in emotional tolerance as mindfulness training.

Another clinical consideration involves patients with chronic medical problems. Both mindfulness and relaxation interventions have shown efficacy for reducing subjective distress among patients with chronic medical conditions (eg, cancer, cardiovascular disease).^29,54,73,93–96^ Thus, either approach may be helpful and could include education about (1) the relationship between stress and disease and (2) the physiological benefits of eliciting the RR in order to increase patient motivation and engagement, perceived control, and self-efficacy.^49^ Clinicians should be cognizant to not suggest that patients have caused their illness through poor stress management or overstate the clinical impact of the RR by describing it as a panacea. Rather, clinicians should emphasize that eliciting the RR is one way patients can take an active role in their health care and improve their overall well-being.

Clinicians might also consider offering both approaches but with attention toward carefully framing their different intentions and utilities. The acceptance-change dialectical emphasized in DBT could provide a useful framework.^70^ This dialectic states that both acceptance and change are important strategies for responding to life events, and in any given situation there may be specific aspects for which one approach is more useful, such that ultimately both are applied. Clinicians might teach patients about the benefits of changing internal and external events when possible and appropriate (ie, a relaxation approach) versus accepting experiences when necessary (ie, a mindfulness approach). Providing training in both strategies could help patients develop a breadth of skills so that they may flexibly choose which is most useful in a given situation. Mindfulness practices can be used to intentionally practice sitting with discomfort to increase distress tolerance and self-efficacy, while relaxation practices can become skills individuals use to practice self-care and cope with acute stressors.

An important consideration that has not received significant empirical attention involves the phenomenon of relaxation-induced anxiety.^97^^,^^98^ Relaxation-induced anxiety involves acute increases in anxiety as a result of relaxation and has been shown to occur among 15% to 54% of individuals, particularly those with anxiety disorders.^97^^,^^99^ It is thought to result from hypervigilance and fears of the effects of relaxation, such as physical sensations (eg, heaviness, warmth) and perceived loss of control.^100^ It may also result from active efforts to control physiological functioning by eliciting a relaxed state.^98^ Relaxation-induced anxiety undermines the efficacy of relaxation treatments and increases treatment attrition,^101^^,^^102^ but it does not need to be a contraindication for mind–body interventions.^103^ For individuals who find relaxation to be frightening or threatening, identifying and exploring the reasons for these fears would be a useful first step. For those who report anxiety around the pressure to feel relaxed, a mindfulness approach might be a useful starting point by reducing this pressure. However, there is a lack of research on relaxation-induced anxiety and how it relates to specific mind–body practices, and similar adverse events may also occur during meditation practices.^104^

### Implications for Research

A primary research implication is the importance of carefully considering whether relaxation is the appropriate control group for mindfulness interventions. Mindfulness interventions have received a surge of research attention over the past 20 years, and relaxation interventions are often used as the comparison group to control for time and attention; however, given the similar benefits of both approaches, and overlap with multimodal interventions, a relaxation intervention may be too strong or otherwise inappropriate as a mindfulness control group. Comparative effectiveness research that aims to directly compare mindfulness and relaxation, however, is a separate issue; here, this comparison would be appropriate but with an emphasis on specifically understanding the relative effects of these 2 approaches, rather than using one as a comparison to evaluate the other. Researchers should carefully consider their theory regarding the effects and mechanisms of mindfulness training for their outcome of interest, and whether relaxation training is indeed an appropriate control. If not, other options for active control groups might include health education training or group support, but identifying an optimal active control group in behavioral research is a complex issue and depends on specific research questions and hypotheses.

Another key consideration for future research is how to integrate mindfulness and relaxation training in a way that maximizes the benefits of both approaches. Offering both approaches as complementary practices could produce the most robust clinical improvement. As mentioned earlier, many multimodal mind–body interventions exist and show efficacy for a variety of problems (eg, SMART-3RP, CBSM). Future research could compare the feasibility, acceptability, and efficacy of multimodal mind–body interventions with those that aim to be primarily mindfulness-based or relaxation-based. Given that relaxation and mindfulness share some overlap, making these comparisons somewhat challenges, researchers could also consider dismantling studies to test active components of interventions that teach multiple skills. It is likely that the feasibility, acceptability, and active ingredients of mind–body interventions would vary across patient populations and characteristics, and thus researchers should consider how clinical factors may moderate each of these factors.

More research is needed to carefully explore the similarities and differences between mindfulness and relaxation interventions in terms of outcomes and mechanisms. For example, whether mindfulness and relaxation can ultimately produce similar improvements in nuanced emotional processes and how intervention dose may moderate these effects would help to inform the theoretical understanding of these approaches and their benefits for specific patient populations. Research to identify predictors of responses to mindfulness and relaxation could also help to tailor mind-body intervention delivery based on patient characteristics (eg, relaxation-induced anxiety). Underlying all of this work is the need for researchers to clearly describe their intervention as relaxation-based or mindfulness-based and deliver the intervention components appropriately in the way that aligns with the meaning and intentions of these practices. It is possible that some of the literature on mindfulness and relaxation interventions is mixed due to discrepancies in the way these interventions are described versus delivered. Lastly, as the purpose of this article is to provide an introductory overview to generate scientific study and discussion, a systematic review of the literature comparing mindfulness and relaxation is beyond the scope of this work; future systematic reviews or meta-analyses in this area would be useful.

## Conclusion

Mindfulness and relaxation are similar yet distinctly different approaches that may have unique benefits and applications depending on specific patient goals, characteristics, and indications. Further research is needed to elucidate the differential effects, mechanisms, predictors, and necessary dose of each approach—whether individually or in combination—for improving diverse health outcomes. We hope that the perspective offered here generates further questions, discussion, and research endeavors in these areas to continue to advance mind–body medicine as an evidence-based approach for optimal wellness.
